# Disaccharide Type Affected Phenolic and Volatile Compounds of Citrus Fiber-Blackberry Cream Fillings

**DOI:** 10.3390/foods10020243

**Published:** 2021-01-26

**Authors:** Josipa Vukoja, Ivana Buljeta, Ivana Ivić, Josip Šimunović, Anita Pichler, Mirela Kopjar

**Affiliations:** 1 Faculty of Food Technology Osijek, F. Kuhača 18, Josip Juraj Strossmayer University in Osijek, 31000 Osijek, Croatia; jjosipa.vukoja@gmail.com (J.V.); ibuljeta@ptfos.hr (I.B.); iivic@ptfos.hr (I.I.); anita.pichler@ptfos.hr (A.P.); 2 Department of Food, Bioprocessing and Nutrition Sciences, North Carolina State University, Raleigh, NC 27695, USA; simun@ncsu.edu

**Keywords:** citrus fiber, blackberries, disaccharides, phenols, color, volatiles

## Abstract

The food industry is continuously developing ingredients, processing methods and packaging materials to improve the quality of fruit products. The aim of this work was to study the possibility of using citrus fiber in the preparation of blackberry cream fillings in combination with disaccharides (sucrose, maltose and trehalose). Evaluations of the phenolics, proanthocyanidins, antioxidant activity, color and volatiles of blackberry cream fillings were conducted after preparation and after three months of storage. Blackberry cream fillings were prepared from citrus fiber (5%), blackberry juice and disaccharides (50%). Disaccharide type had an effect on all investigated parameters. The highest phenol content was in fillings with trehalose (4.977 g/100 g) and the lowest was in fillings prepared with sucrose (4.249 g/100 g). The same tendency was observed after storage. Fillings with maltose had the highest proanthocyanidins content (473.05 mg/100 g) while fillings with sucrose had the lowest amount (299.03 mg/100 g) of these compounds. Regarding volatile compounds, terpenes and aldehydes and ketones were evaluated in the highest concentration. Terpenes were determined in the highest concentration in fillings with trehalose (358.05 µg/kg), while aldehydes and ketones were highest in fillings with sucrose (250.87 µg/kg). After storage, concentration of volatiles decreased. These results indicate that the selection of adequate disaccharides is very important since it can influence the final quality of the product.

## 1. Introduction

Cream fillings can be used for the preparation of bakery products and different types of confectioneries. Their quality has great impact on the overall quality of the final product, and thus it is necessary to find suitable formulations for this semi-product. In this study, we used citrus fibers, blackberry juice and disaccharides as initial ingredients for cream filling formulation. Nowadays, the importance of healthy diets is of increasing interest for consumers, especially since more and more studies are linking plant-based foods with a lowered occurrence of various types of degenerative diseases. Therefore, one of the main goals of the food industry is ensuring attractive and healthy foods for consumers. Through the years, phenolic compounds and fibers have been put forward as functional plant compounds responsible for the prevention of many diseases and the enhancement of the health state of our organisms [1]. Dietary fibers have been highlighted as compounds responsible for various health benefits, such as decreased risk of development of coronary heart disease, hypertension, diabetes, obesity and some gastrointestinal disorders [2]. On the other hand, polyphenols were subject of numerus studies over the years and put forward as compounds with antioxidant, anti-inflammatory, antimicrobial and antiproliferation activity. Additionally, they can be responsible for the reduction of diverse chronic diseases, such as cardiovascular and neurodegenerative diseases, certain cancers, type II diabetes and osteoporosis [3,4,5,6,7]. Citrus fiber has many food applications in baked products, meats, dairy products, sauces and dressings due to their properties, such as high internal surface area, water holding capacity and apparent viscosity [8,9]. As a source of phenolic and volatile compounds, blackberries were chosen. Blackberries are a rich source of phytochemicals, which strongly influence their quality and contribute to their organoleptic attributes and nutritional value. Phenolics are one of the main groups of phytochemicals present in blackberries with substantial quantities of anthocyanins, proanthocyanidins, flavonols and flavan-3-ols [10,11,12]. These compounds are well known for their health benefits [11,12]. Additionally, these fruits are well known for their specific, pleasant flavor [13,14]. The effect of sweeteners on fruit product quality during processing and storage has been the subject of many studies [15,16,17,18,19,20,21,22,23,24,25,26,27,28,29]. Chemical isomers sucrose, maltose and trehalose were chosen for this study. Sucrose was chosen as a commonly used saccharide in fruit product formulation and maltose and trehalose were selected due to their lower sweetness than sucrose. Additionally, trehalose showed a substantially reduced cariogenic potential [30] compared to sucrose so it is adequate for the formulation of “kind to teeth” and “tooth-friendly” products, which are especially beneficial for children, but does not have the laxative effects of other low-cariogenic bulk sweeteners. Furthermore, trehalose is digested more slowly so it has a lower glycemic index, with a lower insulin release than sucrose [31].

The aim of this study was to prepare cream fillings based on citrus fibers and blackberry juice with the addition of disaccharides, namely sucrose, maltose and trehalose. The influence of type of disaccharide on phenolics, proanthocyanidins, antioxidant activity, color and volatile compounds was investigated. Obtained cream fillings were stored at room temperature for three months in order to evaluate the stability of formulated semi-products.

## 2. Materials and Methods

### 2.1. Materials

Hydrochloric acid, acetic acid, methanol, sodium carbonate, iron chloride, ammonium acetate and Folin–Ciocalteu reagent were purchased from the manufacturer Kemika (Zagreb, Croatia). Trolox was purchased from Sigma (Darmstadt, Germany). 2,2’-azinobis (3-ethylbenzothiozolinesulfonic acid) (ABTS) and 2,2-diphenyl-1-picrylhydrazyl (DPPH) were purchased from Fluka (Darmstadt, Germany). 2,4,6,-tri (2-pyridyl)-s-triazine (TPTZ), gallic acid, procyanidin B2 and myrtenol were purchased from Sigma, Germany. Neocuproine and copper chloride are products of Gram-mol Zagreb, Croatia). Sucrose was purchased from Gram-mol (Zagreb, Croatia), and maltose and trehalose were obtained from Hayashibara doo (Nagase group, Tokyo, Japan). Citrus fibers were obtained from Biesterfeld AG (Zagreb, Croatia).

### 2.2. Preparation of Blackberry Cream Fillings

A mixture of blackberry juice and citrus fiber (5%) was first mixed on a heated magnetic stirrer (80 °C) for 10 min, and then after the mixture was well homogenized, a disaccharide was added in an amount of 50% (sucrose, trehalose or maltose) and the mixture was homogenized for 5 min. The hot mixture was poured into heated 40 mL jars and sealed. One set of samples was used for evaluation of defined parameters immediately after preparation, and the other set of samples was stored for 3 months at room temperature to detect changes during the storage. 

### 2.3. Extraction of Phenols

Extracts of cream fillings were prepared for evaluation of total phenolics, proanthocyanidins and antioxidant activity. For extraction, 2 g of fillings were weighed and 20 mL of acidified methanol (HCl:methanol ratio was 1:99) was added. The homogenized mixture was left for 24 h and then the mixture was filtered, and the resulting extract was used in further analyses.

### 2.4. Determination of the Total Phenolics

The total phenolic content of the samples was determined by modified Folin–Ciocalteu method. Briefly, 0.2 mL of obtained extract was taken and mixed with 1.8 mL of deionizer water, followed by the addition of 10 mL (1:10) Folin–Ciocalteu reagent and 8 mL of sodium carbonate (7.5%) [32]. Mixtures were kept in the dark at room temperature for 120 min to develop the color after which a spectrophotometer (Cary 60, UV–VIS, Agilent Technologies, Santa Clara, CA, USA), set to 765 nm, was used to read the absorbance. All samples were measured in triplicate and the obtained values were interpolated on a gallic acid calibration curve. The results represented g of gallic acid equivalents per 100 g of cream filling (g GAE/100 g). 

### 2.5. Determination of Total Proanthocyanidins

Total proanthocyanidins (PAC) concentration was determined colorimetrically using the dimethylaminocinnamaldehyde (DMAC) method as previously described by Prior et al. [33]. Next, 0.1 mL of the sample was mixed with 0.4 mL of water and 1 mL of 4-dimethyl-amino-cinnamaldehyde solution. After 30 min, absorbance was measured at 640 nm. Results were calculated from the calibration curve of procyanidin B2 so concentration of PAC was expressed as milligrams of procyanidin B2 equivalent per 100 g of cream filling (mg B2E/100 g). Measurements were done in triplicate.

### 2.6. Determination of Antioxidant Activity

The ABTS assay was performed according to the procedure described by Arnao et al. [34] with slight modifications. In a glass tube, 0.1 mL of extract was added and mixed with 3 mL of ABTS reagent. Samples were left in the dark for 95 min and then absorbance was read at a wavelength of 734 nm. Further, the radical scavenging activity method, using 2,2-diphenyl-1-picrylhydrazyl radical (DPPH), was applied to evaluate the antioxidant activity of samples. The method was performed according to instructions previously described by Brand-Williams et al. [35], with some modifications. To a glass tube containing 0.2 mL of extract, 3 mL of DPPH solution was added. Thereafter, the mixture was stirred, left in the dark for 15 min and its absorbance at 517 nm was recorded. The next method used to assess the antioxidant activity of the samples was the Copper (II) reducing antioxidant activity assay (CUPRAC) described by Apak et al. [36]. Copper chloride, neocuproine and ammonium acetate buffer (pH 7) solution were mixed in a ratio of 1:1:1. Then to the obtained mixture, 0.2 mL of extract was added followed by 0.9 mL of distilled water. After 30 min of incubation at room temperature absorbance was recorded at 450 nm. The last method used to assess antioxidant activity was the ferric reducing ability of plasma (FRAP) method described by Benzie and Strain [37] (with some modifications). The mixture, consisting of 0.2 mL of extract and 3 mL of FRAP reagent, was incubated for 30 min in the dark after which the absorbance was measured at 593 nm. For blank, in each method, extract was replaced with water and all measurements were done in triplicate. Antioxidant activity evaluated by ABTS, DPPH, FRAP and CUPRAC method was calculated from the calibration curve with Trolox as standard and expressed as nmol TE/100 g except for ABTS which was expressed as µmol TE/100 g.

### 2.7. Color Measurement and Color Change

Color parameters (L*, a*, b*, °h and C*) of citrus fiber-blackberry cream fillings were measured with a chromometer Minolta CR-400 (Minolta; Osaka, Japan), and Lab system was used to evaluate color. Parameter L* describes the lightness of the samples; i.e., if its value is 0 it means that the sample is black and the value 100 represents a white sample. When the value of the parameter a* or b* is positive, it is a red or yellow sample, and when it is negative, it indicates a green or blue sample, respectively. Parameter C* indicates the color saturation value-chroma and °h is the hue angle. The total color change of samples was calculated according to the following formula:

$$\Delta E={\left({\Delta L}^{*2}+{\Delta a}^{*2}+{\Delta b}^{*2}\right)}^{1/2}$$

### 2.8. Volatile Compounds Analysis

Volatile compounds from the citrus fiber-blackberry cream fillings were determined by gas chromatography/mass spectrometry (GC/MS) (Agilent Technologies, Santa Clara, CA, USA). First of all, it was necessary to extract the volatile compounds from the samples and this was carried out using solid-phase microextraction (SPME). Next, 5 g of the sample and 1 g of NaCl were added to a glass vial (10 mL) and heated on a magnetic stirrer at 40 °C at 300 rpm for 45 min. During this time, volatile compounds from the sample were adsorbed on the SPME fibre coated with divinylbenzene/carboxen/polydimethylsiloxane sorbent (50/30 µm, StableFlex^TM^, Supelco, Bellefonte, PA, USA). In the next step, SPME fiber was inserted into the GC injector where volatiles were desorbed at 250 °C. An HP capillary column (60 m × 0.25 mm × 0.25 μm) was used, and the following method was applied: the initial temperature of 40 °C of oven was held for 2 min, then 6 °C/min up to 230 °C. Helium (He) 5.0 (purity 99.999%) was used as the carrier gas at a flow rate of 1 mL/min at 40 °C. The volatile compounds were identified using a mass selective detector. The temperatures of MS Source and MS Quad were set at 230 °C and 150 °C, respectively. Mass range (m/z) was from 45 to 450 and the ionization energy 70 eV. Compounds were confirmed by matching their mass spectra with the National Molecules 2020, 25, 2624 20 of 23 Institute of Standards and Technology mass spectral database (NIST, East Amwell Township, NJ, USA) and through retention time (RT) and retention index (RI) (Table 1). Two repetitions were conducted for each sample. Quantification was conducted by myrtenol as an internal standard and results were presented as µg/kg.

### 2.9. Water Activity Measurement

Water activity was measured by water activity meter (Pawkit, Washington, DC, USA).

### 2.10. Statistical Analysis

Results were expressed as the mean values ± standard deviation. The obtained results were compared by analysis of variance (ANOVA) and Fisher’s least significant difference (LSD) with the significance defined at *p* < 0.05. In addition, cluster analysis was conducted. For that purpose, joining (tree cluster) mode was used with a single linkage amalgamation rule. All statistical analyses were carried out using the software program STATISTICA 13.1 (StatSoft Inc., Tulsa, OK, USA).

## 3. Results

### 3.1. Total Phenolic and Total Proanthocyanidin Content

The results of the total phenolics and total proanthocyanidins of citrus fiber-blackberry cream fillings (CFB) after preparation and after storage for 3 months at room temperature are presented in Table 1. From these results, it can be established that the type of disaccharide had an effect not only on the content of total phenolics and total proanthocyanidins, but also on the retention of these components during storage. The highest total phenolic content was in the sample with the addition of trehalose (4.977 g/100 g), followed by the sample with the addition of maltose (4.495 g/100 g), and the lowest was in the sample with the addition of sucrose (4.249 g/100 g). After storage, phenol content decreased, but the same trend was maintained; i.e., the sample with trehalose had the highest content of total phenolics (4.758 g/100 g), followed by the sample with maltose (4.152 g/100 g), and the lowest content was in the sample with the addition of sucrose (3.715 g/100 g). Throughout storage of 3 months at room temperature, the highest retention of total phenolics were determined in samples with trehalose 95% and the lowest in samples with added sucrose (87%).

Total proanthocyanidins had a different trend then total phenolics. The highest content of total proanthocyanidins was in the sample with maltose (473.05 mg/100 g), followed by the sample with trehalose (422.40 mg/100 g), and the lowest was in the sample with sucrose (299.93 mg/100 g). After storage, degradation of total proanthocyanidins also occurred, but to a much higher extent. Retention of total proanthocyanidins was highest in the sample with sucrose (24%), and for the samples with maltose and trehalose retention was 15% and 19%, respectively.

### 3.2. Antioxidant Activity 

Antioxidant activity was determined using DPPH, ABTS, FRAP and CUPRAC methods. DPPH method is the most common method for in vitro antioxidant activity evaluation, and it is based on free radical scavenging activity. ABTS method is also based on blocking of free radicals, while FRAP and CUPRAC methods are used for measurement of the ability of antioxidants to reduce ferric iron and cupric ion, respectively [38]. Since different mechanisms are involved in these in vitro evaluation methods, different results were obtained by application of each method. The results of the antioxidant activity of citrus fiber-blackberry cream fillings after preparation and after storage are presented in Table 2. The antioxidant activity determined using the DPPH method was highest in the sample with the addition of maltose (170.95 nmoL TE/100 g), followed by the trehalose sample (168.22 nmoL TE/100 g), and the sucrose sample had the lowest value of antioxidant activity (164.30 nmoL TE/100 g). After storage, antioxidant activity decreased and the trend changed, so the trehalose sample had significantly higher (153.30 nmoL TE/100 g) antioxidant activity than the remaining two samples (139 nmoL TE/100 g). By ABTS method, the sample with sucrose had the highest antioxidant activity (2.786 µmoL TE/100 g) while the remaining two samples had the same antioxidant activity (2.58 µmoL TE/100 g). After storage, a decrease of antioxidant activity was observed. The trehalose sample had the lowest (0.828 µmoL TE/100 g) antioxidant activity while the remaining two samples had the same and higher antioxidant activity (1.115 µmoL TE/100 g). Using FRAP method, samples with sucrose and maltose had the same (224 nmoL TE/100 g) antioxidant activity and higher compared to the sample with trehalose (219.69 nmoL TE/100 g). After storage, lower values were determined compared to the initial samples, so the sample with sucrose had the highest (194.19 nmoL TE/100 g) antioxidant activity and the sample with trehalose had the lowest (178.07 nmoL TE/100 g). Antioxidant activity evaluated by CUPRAC method followed the order of sucrose > trehalose > maltose with values of 181.34 nmoL TE/100 g, 173.01 nmoL TE/100 g and 169.58 nmoL TE/100 g, respectively. After storage, the trend changed and the sample with sucrose had the lowest (110.43 nmoL TE/100 g) antioxidant activity while the sample with trehalose had the highest (127.52 nmoL TE/100 g).

### 3.3. Color Parameters

L*, a* and b* values were evaluated by chromameter and the results are presented in Table 3. The L* value is used to determine whether an object is dark or light. All three samples had lower L* values, so they were relatively dark. There was no difference between the samples with the addition of sucrose and trehalose, while the sample with the addition of maltose was slightly darker compared to the already mentioned two samples. During storage, the L* value increased, so samples were lighter. The L* value increased in the direction of sucrose < maltose < trehalose; i.e., the filling with the addition of sucrose was the darkest and the filling with the addition of trehalose was the lightest. After the preparation of the samples, the parameter a*, which defines the red color, increased in the direction of sucrose < maltose < trehalose; i.e., the filling with the addition of sucrose had the least pronounced red color and the filling with the addition of trehalose had the most. During storage, there was a significant decrease in the a* value; i.e., the loss of red color occurred, which was also visible to the eye. After storage, samples with the addition of sucrose and trehalose had equal a* values, but higher values compared to the sample prepared with the addition of maltose. b* value defines yellowness, and all samples had a positive value. After preparation of samples, the b* value increased in the direction of sucrose < maltose < trehalose. After storage, the trend changed so the sample with the addition of maltose had the highest b* value (and the lowest a*) and the samples with the addition of sucrose and trehalose had a lower b* value (and a higher a*). Based on the L*, a* and b* values, the total color change after storage was calculated. In all samples, the total color change was determined in values that confirm that the color change was visible to the eye. The highest total color change was found in the fillings prepared with maltose (7.61) and the lowest was found in the filling with added sucrose (6.19). Since sucrose is commonly used in the food industry for the preparation of gel-based products, unlike maltose and trehalose, the total color change of the samples with these disaccharides compared to the sample with sucrose was also calculated. This overall color change was very small. For the sample with the addition of trehalose it was 0.99 and for the sample with maltose it was 0.60. After storage, a different trend was observed: the color change for the sample with the addition of trehalose was 0.88 and for the sample with the addition of maltose 1.13. 

In addition to the L*, a* and b* values, the saturation (C*) and color tone (°h) were determined on the chromometer (Table 3). After sample preparation, the C* value increased in the sucrose < maltose < trehalose direction; i.e., the filling with added sucrose had the lowest value and the filling with added trehalose had the highest. During storage, C* decreased, with the highest value being found in the sample with the addition of maltose and the lowest in the sample with the addition of trehalose. The color tone or °h value was the highest for the sample with the addition of trehalose and the lowest for the sample with the addition of sucrose. After storage, there was a significant change in color tone, i.e., the values increased significantly. The largest increase in °h was found for the sample with the addition of maltose (48.18), while the samples with the addition of trehalose and sucrose had significantly lower values of color tone (40.8).

Additionally, the water activity of cream filling was evaluated (Table 4). From the results, it can be seen that water activity values were very high for all samples after preparation and after storage.

### 3.4. Volatile Compounds

Volatiles determined in blackberry juice and citrus fiber-blackberry cream fillings together with their molecular weights, hydrophobicity, vapor pressure and odor description are presented in Table 5. 

Nineteen volatile components were identified in the juice and twenty-eight in the fillings. The volatile compounds were divided into four groups: alcohols, acids, aldehydes and ketones, and terpenes. The main classes were terpenes (D-limonene, guaiacol, linalool, trans-verbenol, nerol, phellandral, α-ionone, γ-ionone, β-ionone) then aldehydes and ketones (hexanal, heptanal, 2-heptenal, 1-octen-3-one, 6-methyl-5-hepten-2-one, octanal, 2-octenal, 2-nonenal, decanal, 2,4-nonadienal, 2-decenal, geranylacetone, lily aldehyde), alcohols (2-ethylhexanol, benzyl alcohol, 1-octanol, phenethyl alcohol, perillyl alcohol) and acids (hexanoic acid and nonanoic acid). Citrus fiber and type of sugar had an impact on the concentration of volatiles in blackberry cream fillings. The results of individual volatile concentrations in blackberry cream fillings after preparation and after storage are presented in Table 6. 

Terpenes were the most abundant group of components in the samples. The trehalose sample had the highest terpene concentration (358.1 µg/kg), while the sucrose sample had the lowest concentration of these compounds (281.9 µg/kg). D-limonene was found in the highest concentration in fillings (293.81 µg/kg), and compared to other terpenes, its concentration was significantly higher. A comparison of the fillings showed that the concentration of limonene decreases in the direction of trehalose > maltose > sucrose. After storage, concentration of limonene decreased. Fillings with trehalose and maltose had the same concentration of these volatiles while the fillings with sucrose had a lower concentration of limonene. Phellandral was a terpene that was not identified in the juice but was identified in fillings. A slightly higher concentration of this terpene was found in the sucrose filling, while the remaining two fillings had same concentration. After storage, there was a decrease in the concentration of this terpene, and the highest concentration had fillings with sucrose and trehalose. The concentration of guaiacol decreased in the direction of sucrose > trehalose > maltose. After storage, the concentration of guaiacol decreased, but the difference between the fillings was not determined. The concentration of linalool after preparation and after storage of the fillings followed the trend of guaiacol. Trans-verbenol was found in the highest concentration in the sucrose filling while the remaining two fillings had the same concentration. This terpene was found to be relatively stable during storage. The concentration of nerol decreased in the direction of sucrose > trehalose > maltose and the same trend was maintained after storage. α-ionone and β-ionone had the same trend after the preparation of fillings; i.e., the highest concentration was found in fillings with the addition of sucrose and trehalose, while after storage a different trend was found. The concentration of α-ionone was the same in all samples, and the concentration of β-ionone decreased in the direction of trehalose > sucrose > maltose. After preparation of the fillings, the concentration of γ-ionone was slightly higher in the fillings with sucrose, while the remaining two samples had almost the same concentration. After storage, the filling with trehalose had the highest concentration of this terpene.

Next to terpenes, aldehydes and ketones were evaluated in the highest concentration. The overall concentration of this group of compounds was the highest in fillings with sucrose and lowest in fillings with maltose. During storage the same tendency remained. Thirteen aldehydes and ketones were detected in the blackberry cream fillings. A few of the aldehydes and ketones were not detected in the blackberry juice (hexanal, 2-heptenal, 1-octen-3-one, 6-methyl-5-hepten-2-one, octanal, 2-nonenal and decanal). Among all aldehydes, hexanal, with green-like note, was determined in the highest amount (54.81–74.05 µg/kg). The highest concentration of these volatile compounds was found in the fillings with the addition of sucrose. After storage, the concentration of hexanal decreased in the direction of sucrose > trehalose > maltose. Fillings with trehalose had the highest concentration of these two volatile compounds (3.25 µg/kg of heptanal and 35.33 µg/kg of 2-octenal). After storage, heptanal was detected only in the filling with maltose. Fillings with sucrose had the highest concentration of 2,4-nonadienal, 2-decanal, geranyl acetone and lily aldehyde. After storage for three months these groups of volatiles slightly decreased. Considering the alcohol group, five alcohols were identified, namely 2-ethylhexanol, benzyl alcohol, 1-octanol, phenethyl alcohol and perillyl alcohol. Benzyl alcohol was detected only in blackberry cream fillings while other alcohols were detected in blackberry juice and in blackberry cream fillings as well. All alcohols, except perillyl acohol, had the highest concentration in the filling with the addition of sucrose. During storage, concentration of 2-ethylhexanol and 1-octanol increased while concentration of benzyl alcohol, phenethyl alcohol and perillyl alcohol decreased. For acids (hexanoic and nonanoic acid), the samples with trehalose and sucrose had equal concentrations (24 µg/kg) while the sample with maltose had a slightly lower acid concentration (21 µg/kg). After storage, the acid was found in the highest concentration in the sample with trehalose (25.6 µg/kg) and in the lowest in the sample with sucrose (20.9 µg/kg).

**Figure 1:**
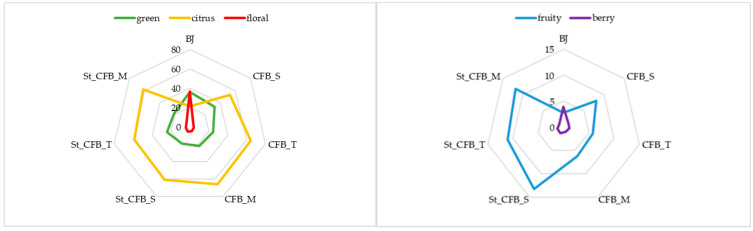
Amount of volatiles of specific flavor profiles of blackberry juice and blackberry cream fillings with the addition of sucrose, maltose and trehalose after preparation and after storage for three months (CFB—citrus fiber-blackberry cream fillings; S—sucrose; M—maltose; T—trehalose; St—storage).

Analyses of the volatiles of specific flavor notes revealed that disaccharides and citrus fiber had an impact on the overall flavor profile of the obtained samples (Figure 1). Other studies on citrus flavors revealed that main components of citrus flavor were limonene (predominant one), β-pinene, linalyl acetate γ-terpinene and citral [39,40]. In our study, citrus flavor note (D-limonene, linalool, nerol, 2-ethylhexanol, 6-methyl-5-hepten-2-one) was the dominant one. The highest amount of these volatiles was in the samples with maltose and trehalose after preparation (66% and 64%, respectively). After storage, the highest retention of citrus volatiles was also in the samples with maltose, 40% higher than in blackberry juice. Green flavor notes (1-octanol, hexanal, heptanal, 2-heptenal, octanal, 2-nonenal, guaiacol) were the highest in the blackberry juice, followed by the blackberry filling with the addition of sucrose. After storage, green volatiles were the highest in the filling with the addition of trehalose. Floral flavor notes (phenethyl alcohol, decanal, geranylacetone, lily aldehyde, phellandral) were determined in higher amounts in blackberry juice (36%) than in the blackberry fillings where their amount after preparation and after storage was under 5.5%. Fruity flavor notes (benzyl alcohol and 1-octen-3-one) were higher in the samples after storage for three months than in the samples after preparation. The highest amount of fruity volatiles was in the samples with the addition of sucrose (after preparation (8%) and after storage (13%)). The amount of berry flavor note (α-ionone, γ-ionone, β-ionone) was very small in the blackberry juice (3.9%) as well as in the blackberry cream fillings after preparation and after storage (under 1.5%). 

**Figure 2:**
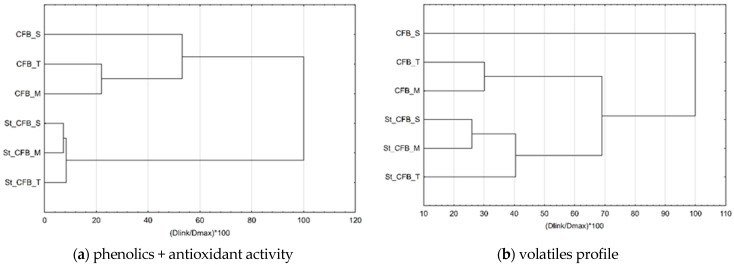
Cluster analysis of similarity of samples according to phenolics and antioxidant activity (**a**) and volatiles profile (**b**) (CFB—citrus fiber-blackberry cream fillings; S—sucrose; M—maltose; T—trehalose; St—storage).

According to the similarity test of cream fillings (Figure 2), it can be seen that fillings with maltose and trehalose were grouped together when phenolics and antioxidant activity were taken as grouping parameters as well as when grouping parameters were volatiles profile. After storage, this similarity changed and fillings with sucrose and maltose showed higher similarity according to both grouping parameters.

## 4. Discussion

The aim of this study was the formulation of cream fillings enriched with fibers, blackberry volatiles and phenolics. Cream fillings were prepared from citrus fiber, blackberry juice and disaccharides in order to investigate the influence of disaccharide type on volatiles, phenolics, antioxidant activity and color. Generally, water has a very specific and important role as a solvent and a reactant in many chemical reactions in foods. Our cream fillings had high water activity and thus reactions limited by diffusion were enhanced in all filings. Those reactions together with oxidation and molecular mobility can cause degradation of sensitive compounds [41] such as volatiles and phenolics. In order to minimize those degradation reactions throughout preparation as well as storage, different types of disaccharide (sucrose, maltose and trehalose) were used. 

The positive effect of trehalose on the retention of terpenes during the preparation of cream fillings as well as during storage was in accordance with other studies on the effect of saccharide addition on the volatile compounds in different fruit products [15,16,17,18,19,20,21,22]. In those studies, it was emphasized that the positive effect of trehalose on volatiles depended on the properties of the volatile compounds, but also on the sample composition and conditions during processing and storage [15,16,17,18,19,20,21,22]. The evaluation of fruity esters and other volatiles responsible for strawberry flavor in strawberry cream filling showed that their retention during preparation (evaporation and freeze-drying) highly depended on process conditions and trehalose amount (3, 5 and 10%). Throughout this study, it was determined that there was no increase in the retention of fruity esters with the increase of trehalose amount [18,21]. It has been proven that disaccharide type, namely sucrose, maltose and trehalose, can effect a tart cherry flavor profile, each of them contributing in a different way to some specific flavor notes [22].

Also, there have been studies showing similar results on the protection of phenolics, flavonoids and anthocyanins by trehalose addition under different conditions during processing and storage [21,22,23,24,25,26,27,28,29]. The impact of sucrose, maltose and trehalose in freeze-dried tart cherry puree was investigated by Lončarić et al. [27], and the authors concluded that the type of disaccharide and its amount played an important role in the retention of phenolics, flavonoids and anthocyanins. From the obtained results, it was concluded that addition of maltose and trehalose had a higher positive effect on the preservation of phenolics than sucrose. In addition, antioxidant activity was evaluated. Good correlation between phenolics, flavonoids and anthocyanins was established with values of antioxidant activity obtained with ABTS and FRAP methods, but not with DPPH method [27]. The influence of trehalose on phenolics in orange jelly was shown to be positive but only after 135 days of storage. Actually, after preparation orange jelly prepared with sucrose had higher phenolics content. During storage, samples containing trehalose and those without trehalose decreased in total phenolics content by 9.14% and 27.8%, respectively [29]. In addition, antioxidant activity determined by DPPH and ABTS methods followed the trend observed for phenolics [29]. Study on the stability of phenolics in raspberry cream fillings prepared with different hydrocolloids and with the addition of saccharides (different combinations of sucrose, fructose and trehalose) revealed that samples with trehalose addition had the highest content of phenolics after preparation as well as after 8 and 16 months of storage. The same tendency was observed for antioxidant activity evaluated by DPPH method [28]. In our study, good correlation between antioxidant activity and phenolics couldn’t be established. Structural changes of phenolics such as the formation of polymerized phenols and/or oxidized phenols can occur during preparation and storage of samples; thus, these formed phenolics can exhibit higher or lower antioxidant activity than non-polymerized and non-oxidized phenols [21,42,43,44,45]. Additionally, Maillard reaction occurred during preparation and storage. Maillard reaction products are known as potential antioxidants [46,47] and those reactions depend on the disaccharide type and its stability to hydrolysis, so this could be another reason for not obtaining a good correlation between phenolics and antioxidant activity. Study on the evaluation of intensity of browning reactions in model systems containing sucrose and trehalose revealed that sucrose hydrolysis was noticeable while trehalose hydrolysis was negligible. Consequently, the sucrose-containing system developed brown pigments much faster than the trehalose model system [48]. In addition, it was established that rate constants for formation of brown color were from 200- to 2000-fold greater in the sucrose-containing system than in trehalose one [49]. 

The impact of disaccharides on proanthocyanidins was observed during preparation and their degradation was quite pronounced during the storage period. Similar results were observed in the study by Howard et al. [47]. The authors concluded that the formulation and storage conditions had a great influence on the preservation of proanthocyanidins and their loss. They observed that retention of proanthocyanidins was higher in blueberry sugar-free jams than in sugar jams, and their degradation during storage was more pronounced in sugar jams [47].

The type of disaccharide also affected color parameters, which is in agreement with other studies. Licciardello and Muratore [50] also determined that additives have a large influence on the visible color change of blood orange marmalade during storage. A noticeable visual change of color was also determined when trehalose was added to evaporated or freeze-dried strawberry cream fillings, after preparation of samples and after storage [21]. The same effect was observed on orange jellies with sucrose and trehalose after preparation and during storage [29]. Additionally, Nowicka and Wojdyło [51] investigated the effect of sweeteners on the color of tart cherry puree during storage and found that color parameters depended on the type of sweetener used. 

Generally, fruit matrix composition, conditions during processing and storage affected interactions between all fruit matrix components resulting in retention or loss of volatiles and phenolics. 

The investigated disaccharides were sucrose, maltose and trehalose. These disaccharides are chemical isomers. However, their behavior in a complex matrix can be different than it was case in our study. It is evident from our results that those disaccharides effected chosen parameters differently. Their behavior in a much simpler system like water was also different, so it can be expected that those differences are more pronounced in a complex matrix. Trehalose has higher affinity for water and it can bind to a larger number of water molecules in comparison to maltose and sucrose, leading to its higher effect on water structure. The formation of clusters in water was also one factor that was different for those isomers. Trehalose has the ability to form larger clusters than sucrose, but on the other hand, smaller than maltose. Considering hydration number, radius of gyration, glycosidic dihedral angles and cluster formation, it has been concluded that trehalose/water solutions were more homogeneous than water solutions of sucrose or maltose [52]. Disaccharides also can develop steric hindrance which can slow down the nucleophilic attacks of water on sensitive compounds like phenolics [53]. Investigation of disaccharides’ ability to slow down water dynamics revealed that trehalose induced slightly stronger retardation than other investigated saccharides (sucrose, maltose and glucose). Trehalose affected the single-particle and collective rotation and translation of water to a slightly larger extent than other saccharides [54]. Olsson and Swenson [55] observed that trehalose had a stronger perturbing effect on the structure of water and that water molecules interacted slightly differently with the different atomic sites of the two disaccharides. Additionally, a very important property of trehalose is its high stability regarding hydrolysis in comparison with sucrose [53]. The formation of stable intramolecular complexes between trehalose and unsaturated compounds is also a possible explanation of trehalose’s positive effect on phenolics and some volatiles [56,57,58]. Volatiles are characterized by their diffusion. Sugars have different diffusion coefficients in water and can also change the diffusion coefficient of water [59], so throughout the modification of the diffusion coefficient of volatiles they can have effects on their retention in the fruit product matrix. We can conclude that disaccharides effected water dynamics, resulting in the effect of sugars on volatile and phenolic compounds.

Obtained cream fillings as semi-product can be used in the preparation of bakery products and different types of confectionery. This research is a good starting point for the improvement of existing products and for the development of new ones as well. Future studies should be governed to formulate the final products to investigate the influence of cream fillings on overall quality, such as flavor and color. Additionally, these cream fillings could be used for improvement of the nutritional value of newly produced products through their enrichment with fibers and phenolics, ensuring possible health benefits.

## 5. Conclusions

The results of our research showed that disaccharide type used for the formulation of citrus fiber-based cream fillings had an important impact on both active components that we investigated, phenolics and volatiles. Overall, trehalose had a higher positive impact on volatiles, with a desirable flavor note, as well as a positive impact on phenolics and proanthocyanidins. Both, trehalose and maltose can be used for the formulation of cream fillings that are less sweet than sucrose ones, and additionally trehalose samples can have possible health benefits. 

## Figures and Tables

**Table 1:** Total phenolics (TPC) and proanthocyanidins (PAC) content in citrus fiber-blackberry cream fillings with the sucrose, maltose and trehalose after preparation and after storage for three months.

Samples	TPC (g/100 g)	PAC (mg/100 g)
After preparation		
CFB_sucrose	4.249 ± 0.015 ^c^	299.93 ± 8.72 ^c^
CFB_maltose	4.495 ± 0.029 ^b^	473.05 ± 8.72 ^a^
CFB_trehalose	4.977 ± 0.006 ^a^	422.40 ± 6.54 ^b^
After storage		
CFB_sucrose	3.715 ± 0.018 ^e^	69.511 ± 0.584 ^e^
CFB_maltose	4.152 ± 0.011 ^d^	70.164 ± 0.693 ^e^
CFB_trehalose	4.758 ± 0.019 ^d^	80.221 ± 0.507 ^d^

Means ± standard deviation in the same column with the same letters are not significantly different (*p* ≤ 0.05). CFB—citrus fiber-blackberry cream fillings.

**Table 2:** Antioxidant activity of citrus fiber-blackberry cream fillings after preparation and after storage for 3 months.

Samples	DPPH (nmoL TE/100 g)	ABTS (µmoL TE/100 g)	FRAP (nmoL TE/100 g)	CUPRAC (nmoL TE/100 g)
After preparation				
CFB_sucrose	164.30 ± 0.37 ^c^	2.786 ± 0.029 ^a^	224.49 ± 0.68 ^a^	181.34 ± 1.88 ^a^
CFB_maltose	170.95 ± 0.21 ^a^	2.564 ± 0.018 ^b^	223.94 ± 0.78 ^a^	169.58 ± 1.42 ^c^
CFB_trehalose	168.22 ± 0.13 ^b^	2.597 ± 0.054 ^b^	219.69 ± 0.50 ^b^	173.01 ± 1.24 ^b^
After storage				
CFB_sucrose	139.32 ± 0.61 ^e^	1.114 ± 0.037 ^c^	194.19 ± 0.86 ^c^	110.43 ± 0.91 ^f^
CFB_maltose	138.83 ± 0.54 ^e^	1.118 ± 0.043 ^c^	180.48 ± 0.49 ^d^	120.11 ± 0.83 ^e^
CFB_trehalose	153.30 ± 0.47 ^d^	0.828 ± 0.027 ^d^	178.07 ± 0.25 ^e^	127.52 ± 0.77 ^c^

Means ± standard deviation in the same column with the same letters are not significantly different (*p* ≤ 0.05). CFB—citrus fiber-blackberry cream fillings.

**Table 3:** Color parameters (L*, a*, b*, °h and C*) of citrus fiber-blackberry cream fillings after preparation and after storage at room temperature for three months.

Samples	L*	a*	b*	ΔE	ΔEs	°h	C*
After preparation							
CFB_sucrose	28.64 ± 0.02 ^a^	9.47 ± 0.05 ^a^	2.35 ± 0.05 ^a^			13.95 ± 0.21 ^d^	9.76 ± 0.06 ^c^
CFB_maltose	28.36 ± 0.03 ^b^	9.94 ± 0.02 ^b^	2.59 ± 0.02 ^b^		0.60	14.60 ± 0.11 ^c^	10.27 ± 0.02 ^b^
CFB_trehalose	28.67 ± 0.01 ^a^	10.39 ± 0.01 ^c^	2.72 ± 0.02 ^c^		0.99	14.68 ± 0.11 ^c^	10.74 ± 0.01 ^a^
After storage							
CFB_sucrose	32.10 ± 0.08 ^c^	4.60 ± 0.07 ^d^	3.97 ± 0.06 ^d^	6.19		40.76 ± 0.29 ^b^	6.07 ± 0.02 ^e^
CFB_maltose	32.87 ± 0.02 ^d^	4.17 ± 0.04 ^e^	4.66 ± 0.06 ^e^	7.61	1.13	48.18 ± 0.15 ^a^	6.25 ± 0.07 ^d^
CFB_trehalose	32.96 ± 0.08 ^d^	4.46 ± 0.05 ^d^	3.88 ± 0.06 ^d^	7.41	0.88	40.97 ± 0.13 ^b^	5.91 ± 0.07 ^f^

Means ± standard deviation in the same column with the same letters are not significantly different (*p* ≤ 0.05); CFB—citrus fiber-blackberry cream fillings; ΔE—total color change after storage; ΔEs—total color change of fillings with trehalose or maltose in relation to the filling with sucrose.

**Table 4:** Water activity of citrus fiber-blackberry cream fillings after preparation and after storage at room temperature for three months.

Samples	After Preparation	After Storage
CFB_sucrose	0.94	0.95
CFB_maltose	0.95	0.96
CFB_trehalose	0.96	0.97

**Table 5:** Volatile compounds detected in blackberry juice and citrus fiber-blackberry cream fillings.

Compounds	BJ *	RT ^1^	RI ^2^	MW ^3^	log P ^4^	Vp ^5^	OD ^6^
2-ethylhexanol	+	19.956	1030	139	3.10	0.207	citrus
Benzyl alcohol	−	20.111	1037	192.3	3.2	0.008	fruity
1-octanol	+	22.48	1071	130.2	3.00	0.079	green
Phenethyl alcohol	+	24.6	1103	122.2	1.40	0.087	floral
Perillyl alcohol	+	33.93	1290	152.2	2.10	0.006	woody
Hexanoic acid	−	18.535	1005	116.1	1.90	0.158	fatty
Nonanoic acid	+	33.490	1281	158.2	3.50	0.009	fatty
Hexanal	−	5.132	800	100.2	1.80	10.888	green
Heptanal	+	10.761	897	114.2	2.30	3.854	green
2-heptenal	−	14.953	956	112.1	2.10	1.823	green
1-octen-3-one	−	16.464	982	126.2	2.40	1.063	fruity
6-methyl-5-hepten-2-one	−	17.106	987	126.2	1.90	1.277	citrus
Octanal	−	18.080	998	128.2	2.70	2.068	green
2-octenal	+	21.492	1054	126.2	2.60	0.552	fatty
2-nonenal	−	27.113	1155	140.2	3.10	0.256	green
Decanal	−	29.502	1200	156.3	3.80	0.207	floral
2,4-nonadienal	+	29.859	1205	138.2	2.70	0.102	fatty
2-decenal	+	32.231	1255	154.3	3.70	0.067	fatty
Geranylacetone	+	39.598	1448	194.3	3.834	0.016	floral
Lily aldehyde	+	41.150	1519	204.3	4.216	0.005	floral
D-limonene	+	19.413	1018	136.2	4.57	0.198	citrus
Guaiacol	+	23.166	1080	124.1	1.32	0.179	green
Linalool	+	23.962	1096	154.3	2.970	0.016	citrus
Trans-verbenol	+	25.7163	1129	152.2	1.60	0.033	herbal
Nerol	+	30.631	1222	154.3	3.47	0.013	citrus
Phellandral	−	32.613	1264	152.2	2.70	0.098	floral
α-ionone	+	38.949	1420	192.3	3.995	0.014	berry
γ-ionone	+	40.183	1473	192.3	3.2	0.008	berry
β-ionone	+	40.346	1480	192.3	3.995	0.017	berry

BJ—blackberry juice; ^1^ RT (min)—retention time of volatiles; ^2^ RI—retention index of volatiles; ^3^ MW—molecular weight; ^4^ log P—logarithm of octanol water coefficient that indicates the relative hydrophobicity of compound; ^5^ vp—vapor pressure (mm/Hg); ^6^ OD—odor description; data were obtained from http://www.thegoodscentscompany.com and http://www.chemicalbook.com; * “+” detected volatiles only in blackberry juice and “−“detected volatiles in juice and cellulose/raspberry complexes.

**Table 6:** Volatile compounds (µg/kg) of blackberry juice/blackberry cream fillings with after preparation and after storage.

Compounds	After Preparation			After Storage		
	CFB_S	CFB_M	CFB_T	CFB_S	CFB_M	CFB_T
*Alcohols*	*57.25 ± 0.66*	*44.41 ± 0.69*	*45.30 ± 0.67*	*53.51 ± 0.41*	*49.88 ± 0.39*	*49.31 ± 0.37*
2-ethylhexanol	14.36 ± 0.20 ^d^	9.65 ± 0.31 ^e^	10.45 ± 0.22 ^e^	19.38 ± 0.15 ^a^	17.36 ± 0.02 ^b^	16.83 ± 0.19 ^c^
Benzyl alcohol	17.72 ± 0.14 ^a^	11.76 ± 0.04 ^c^	13.47 ± 0.20 ^b^	7.95 ± 0.06 ^d^	6.54 ± 0.08 ^e^	8.16 ± 0.06 ^d^
1-octanol	15.64 ± 0.03 ^c^	12.58 ± 0.21 ^d^	12.21 ± 0.08 ^d^	17.98 ± 0.06 ^b^	18.25 ± 0.02 ^a^	17.59 ± 0.05 ^b^
Phenethyl alcohol	5.16 ± 0.14 ^b^	6.42 ± 0.07 ^a^	4.95 ± 0.08 ^b^	4.88 ± 0.13 ^b^	4.98 ± 0.20 ^b^	4.10 ± 0.01 ^c^
Perillyl alcohol	4.38 ± 0.15 ^c^	4.00 ± 0.08 ^b^	4.23 ± 0.09 ^a, b^	3.32 ± 0.01 ^c^	2.76 ± 0.07 ^d^	2.64 ± 0.05 ^d^
*Acids*	*23.99 ± 0.24*	*21.65* *± 0.08*	*24.10* *± 0.14*	*20.88* *± 0.05*	*22.22* *± 0.27*	*25.57* *± 0.29*
Hexanoi c acid	21.01 ± 0.23 ^c^	18.06 ± 0.05 ^e^	21.48 ± 0.10 ^c^	18.49 ± 0.02 ^d^	22.22 ± 0.27 ^b^	23.18 ± 0.27 ^a^
Nonanoic acid	2.98 ± 0.02 ^b^	3.59 ± 0.03 ^a^	2.62 ± 0.05 ^c^	2.39 ± 0.03 ^d^	-	2.39 ± 0.02 ^d^
*Aldehydes and ketones*	*250.87 ± 3.14*	*156.98* *± 2.17*	*203.62* *± 3.74*	*214.38* *± 5.16*	*171.69* *± 2.54*	*196.67* *± 4.19*
Hexanal	74.05 ± 0.13 ^a^	46.23 ± 0.32 ^d^	54.81 ± 0.75 ^b^	50.04 ± 1.08 ^c^	34.41 ± 0.15 ^e^	34.48 ± 1.61 ^e^
Heptanal	2.75 ± 0.10 ^b^	2.65 ± 0.01 ^c^	3.25 ± 0.11 ^a^	-	1.63 ± 0.01 ^d^	-
2-heptenal	48.00 ± 0.56 ^a^	16.68 ± 0.03 ^d^	32.79 ± 0.97 ^c^	40.61 ± 1.28 ^b^	14.07 ± 0.35 ^e^	33.79 ± 0.36 ^c^
1-octen-3-one	25.70 ± 0.67 ^c^	19.13 ± 0.14 ^d^	19.00 ± 0.31 ^d^	43.53 ± 1.13 ^b^	48.77 ± 1.04 ^a^	45.86 ± 1.24 ^a,b^
6-methyl-5-hepten-2-one	10.73 ± 0.05 ^c^	9.52 ± 0.11 ^d^	9.51 ± 0.07 ^d^	11.38 ± 0.46 ^b^	11.30 ± 0.13 ^b^	15.57 ± 0.21 ^a^
Octanal	24.09 ± 0.11 ^a^	20.78 ± 0.17 ^d^	21.97 ± 0.08 ^c^	23.77 ± 0.95 ^a,b^	19.80 ± 0.21 ^e^	22.86 ± 0.15 ^b^
2-octenal	34.94 ± 1.14 ^a^	16.44 ± 0.01 ^d^	35.33 ± 0.78 ^a^	20.86 ± 0.01 ^b^	18.28 ± 0.18 ^c^	18.71 ± 0.19 ^c^
2-nonenal	5.57 ± 0.05 ^a^	4.90 ± 0.04 ^b^	5.08 ± 0.12 ^b^	4.34 ± 0.05 ^d^	4.89 ± 0.03 ^b^	4.55 ± 0.06 ^c^
Decanal	7.24 ± 0.14 ^b^	7.29 ± 1.13 ^a,b^	6.44 ± 0.08 ^c^	7.68 ± 0.05 ^a^	7.89 ± 0.17 ^a^	7.69 ± 0.06 ^a^
2,4-nonadienal	5.17 ± 0.10 ^a^	3.62 ± 0.10 ^b^	3.25 ± 0.09 ^c^	2.76 ± 0.08 ^d^	2.45 ± 0.04 ^e^	3.10 ± 0.07^c^
2-decenal	4.77 ± 0.03 ^a^	3.69 ± 0.07 ^c^	4.71 ± 0.06 ^a^	4.00 ± 0.05 ^b^	3.39 ± 0.16 ^c^	3.50 ± 0.14 ^c^
Geranyl acetone	3.97 ± 0.03 ^a^	3.82 ± 0.05 ^b^	3.94 ± 0.16 ^a^	3.12 ± 0.00 ^d^	3.62 ± 0.06 ^b^	3.29 ± 0.03 ^c^
Lily aldehyde	3.88 ± 0.02 ^a^	2.22 ± 0.00 ^c^	3.53 ± 0.16 ^b^	2.29 ± 0.01 ^c^	1.19 ± 0.00 ^d^	3.28 ± 0.07 ^b^
*Terpenes*	*281.91 ± 1.53*	*328.39* *± 1.93*	*358.05* *± 8.01*	*223.44* *± 6.67*	*273.50* *± 4.67*	*271.42* *± 3.01*
D-limonene	240.29 ± 0.89 ^c^	293.81 ± 1.47 ^b^	320.03 ± 7.46 ^a^	189.56 ± 5.68 ^d^	241.51 ± 3.95 ^c^	237.80 ± 2.60 ^c^
Guaiacol	6.97 ± 0.06 ^a^	5.30 ± 0.10 ^d^	6.09 ± 0.10 ^b^	5.22 ± 0.21 ^c, d^	5.21 ± 0.37 ^c, d^	5.72 ± 0.19 ^c^
Linalool	16.26 ± 0.07 ^a^	14.78 ± 0.03 ^c^	15.63 ± 0.02 ^b^	13.83 ± 0.24 ^d^	13.73 ± 0.16 ^d^	13.47 ± 0.04 ^e^
Trans-verbenol	3.41 ± 0.12 ^a^	2.58 ± 0.04 ^c^	2.68 ± 0.03 ^c^	3.12 ± 0.03 ^b^	2.69 ± 0.02 ^c^	2.35 ± 0.01 ^d^
Nerol	3.42 ± 0.18 ^a^	2.60 ± 0.16 ^c^	2.89 ± 0.20 ^b, c^	3.00 ± 0.10 ^b^	2.30 ± 0.08 ^d^	2.79 ± 0.01 ^c^
Phellandral	4.99 ± 0.04 ^a^	4.14 ± 0.03 ^b^	4.39 ± 0.18 ^b^	3.48 ± 0.19 ^c^	3.08 ± 0.03 ^d^	3.35 ± 0.01 ^c^
α-ionone	1.62 ± 0.03 ^a^	0.83 ± 0.01 ^c^	1.68 ± 0.00 ^a^	1.37 ± 0.02 ^b^	1.33 ± 0.02 ^b^	1.38 ± 0.00 ^b^
γ-ionone	2.80 ± 0.13 ^a^	2.52 ± 0.05 ^b^	2.56 ± 0.01 ^b^	2.42 ± 0.19 ^b,c^	2.41 ± 0.03 ^c^	2.91 ± 0.14 ^a^
β-ionone	2.15 ± 0.03 ^a^	1.83 ± 0.02 ^b^	2.10 ± 0.01 ^a^	1.43 ± 0.02 ^d^	1.25 ± 0.00 ^e^	1.65 ± 0.01 ^c^

Means ± standard deviation in the same column with the same letters are not significantly different (*p* ≤ 0.05). CFB—citrus fiber-blackberry cream fillings; S—sucrose; M—maltose; T—trehalose; italic values define sum of specific group of volatiles. Italic values presents total sum of specific group of volatiles.

## Data Availability

Not applicable.
